# Prevalence and control of asthma among US youths aged 12 to 19 years (2001–2023): A cross-sectional study

**DOI:** 10.1097/MD.0000000000049425

**Published:** 2026-06-26

**Authors:** Qi Cui, Zhongyan Xu, Keran Jia, Liyun An, Jun Qiao, Fukun Wang

**Affiliations:** a Clinical Laboratory, Bethune International Peace Hospital, Shijiazhuang, Hebei, China; b Department of Pharmacology, School of Medicine, Southern University of Science and Technology, Shenzhen, China.

**Keywords:** asthma, NHANES, prevalence, self-reported poor control indicators, US youths

## Abstract

Childhood asthma is linked to socioeconomic factors, but prior studies and CDC reports lack 22-year stratified analyses of the 12 to 19 years subgroup, simultaneous assessment of multiple outcomes, and evaluation of post-2012 disparity changes. This age group is a critical transition period for asthma management, with long-term trends and disparities poorly understood. To systematically explore long-term trends in asthma prevalence and self-reported poor control indicators (asthma attacks, ED visits) among US youths aged 12 to 19 years, and sociodemographic disparities over 2001 to 2023. This cross-sectional trend analysis used data from the National Health and Nutrition Examination Survey spanning 22 years (2001–2023), including 17,096 adolescents (3290 with current asthma). Sociodemographic subgroups were defined by age and race/ethnicity. Analyses incorporated combined mobile examination center (MEC) and interview weights to account for National Health And Nutrition Examination Survey’ complex sampling design. A sensitivity analysis excluded the 2017 to 2020 cycle to address COVID-19-related data disruptions. Asthma prevalence remained disproportionately high in non-Hispanic Black youth, with no statistically significant narrowing of disparities post-2012 (2.17% in 2013–2016 vs 3.99% in 2021–2023; *P* > .05). Self-reported poor control indicators were suboptimal overall, with no detectable improvement trends during the study period. Sensitivity analysis (excluding 2017–2020) confirmed the robustness of core findings. Persistent sociodemographic disparities in adolescent asthma burden highlight the need for targeted interventions addressing structural inequities. Future studies should include confounder adjustment to clarify potential causal pathways.

## 1. Introduction

Asthma is a chronic noncommunicable respiratory disease characterized by chronic airway inflammation, reversible airflow limitation, and recurrent episodes of wheezing, shortness of breath, chest tightness, and cough, which imposes a heavy disease burden and public health challenge worldwide.^[1]^ Data from the 2021 Global Burden of Disease (GBD) Study show that the global number of people living with asthma has reached 260 million, with more than 436,000 asthma-related deaths annually. Although the global age-standardized prevalence, incidence, and mortality of asthma all exhibited a downward trend from 1990 to 2021, asthma remains one of the leading chronic diseases contributing to the loss of disability-adjusted life years (DALYs) among children and adolescents.^[1]^ In the United States, asthma is a major chronic disease affecting the health of the population across all age groups. National surveillance data from 2020 to 2021 indicate that the prevalence of asthma in the overall US population is approximately 7.8%, with more than 986,000 asthma-related emergency department (ED) visits, over 94,000 hospitalizations, and 3517 deaths reported annually, placing enormous pressure on the healthcare system and socioeconomic landscape.^[2]^ Previous studies have estimated that the annual asthma-related economic burden in the United States exceeds $810 billion, including direct medical expenditures and indirect productivity losses resulting from missed work and school days.^[3,4]^

Adolescence (aged 10–19 years) is a critical transitional period for physical, psychological, and social behavioral development, as well as a key stage associated with unique challenges for asthma management.^[5]^ Globally, approximately 11% of adolescents are affected by asthma, while the prevalence of asthma among US adolescents has long remained at a high level, and the asthma control rate in this population is significantly lower than that in adults and younger children.^[2,6]^ The core difficulty in asthma management among adolescents is poor treatment adherence. Studies have shown that the adherence to inhaled corticosteroids (ICS) therapy among adolescents monitored via electronic devices is <50%, and suboptimal adherence directly leads to poor asthma control, elevated risk of acute exacerbations, increased frequency of ED visits and hospitalizations, and even higher mortality risk.^[5,7]^ Meanwhile, the comorbidity rate of asthma and psychological conditions such as depression is significantly higher in the adolescent population. Depressive symptoms not only further reduce treatment adherence, but also form a vicious cycle with acute asthma exacerbations and adverse health outcomes, thereby exacerbating the overall disease burden of asthma.^[8]^ In addition, asthma control status during adolescence can directly affect lung function trajectories and the risk of chronic respiratory diseases in adulthood. Therefore, clarifying the epidemic trends and health disparities of asthma in this population is of great importance for the development of life-course asthma prevention and control strategies.^[9]^

Multiple previous studies have conducted surveillance and analysis of asthma trends using nationally representative datasets in the United States, providing critical foundational evidence for understanding the epidemiological characteristics of asthma in the country. Studies based on the Healthcare Cost and Utilization Project (HCUP) National Emergency Department Sample (NEDS) and National Inpatient Sample (NIS) analyzed national and state-level trends in asthma-related ED visits and hospitalizations among the overall US population from 2010 to 2020, revealing disparities in asthma-related healthcare utilization across groups stratified by age, gender, and race/ethnicity.^[2]^ A study based on data from the 2005 to 2020 National Health and Nutrition Examination Survey (NHANES) explored the prevalence trends and influencing factors of polypharmacy among adult asthma patients in the United States, and clarified the impacts of asthma comorbidities and socioeconomic factors on disease management.^[3]^ Multi-state epidemiological studies have also analyzed the acute effects of risk factors such as ambient air pollution on asthma-related ED visits, further elucidating the environmental drivers of asthma onset and acute exacerbations.^[10]^ In addition, multiple studies based on the Youth Risk Behavior Surveillance System (YRBSS) have reported the association between asthma and depression, as well as health-risk behaviors among adolescents, filling the evidence gap in the field of psychological comorbidities of adolescent asthma.^[5,11]^ Although these foundational studies have provided valuable insights into the burden of adolescent asthma, they still lack systematic analyses in 3 core dimensions: (1) long-term (22-year) stratified analyses of the population aged 12 to 19 years, with existing studies failing to disaggregate this group into 12 to 15 years and 16 to 19 years subgroups, thus unable to accurately capture age-specific trend changes; (2) the absence of simultaneous assessment of asthma prevalence, self-reported indicators of poor asthma control, and sociodemographic disparities over a 2-decade time span; and (3) the yet-to-be-clarified evolutionary trend of whether sociodemographic disparities related to adolescent asthma narrowed or widened after 2012. Notably, few previous long-term trend studies have analyzed adolescents aged 12 to 19 years as an independent subgroup, resulting in the long-term changing patterns of asthma prevalence, acute attack rates, ED visit characteristics, and sociodemographic disparities in this population remaining poorly understood.

This study systematically addresses the aforementioned research gaps using nationally representative data from the 22-year (2001–2023) NHANES in the United States. The core analytical contents of this study include: systematic analysis of the long-term temporal trends in asthma prevalence and self-reported indicators of poor asthma control among US adolescents aged 12 to 19 years; stratified analyses of the 12 to 15 years and 16 to 19 years subgroups to clarify differences in the epidemiological and control characteristics of asthma across different stages of adolescence; and dissection of disparities in asthma-related outcomes among adolescents across sociodemographic subgroups, as well as the long-term evolutionary characteristics of these disparities over the 22-year period, with a focus on assessing the direction of changes in health inequalities after 2012.

## 2. Methods

### 2.1. Study design and data source

NHANES is a series of cross-sectional surveys employing a complex, multistage probability sampling design to represent the civilian, noninstitutionalized U.S. population. This study analyzed NHANES data spanning 22 years (2001–2023). Notably, NHANES data collection was paused due to the COVID-19 pandemic from March 2017 to 2020 and resumed in August 2021 (continuing until August 2023). In accordance with NHANES data access guidelines (NHANES, 2024; https://wwwn.cdc.gov/nchs/nhanes/default.aspx), the survey cycles were adjusted to 6 periods for analysis: 2001 to 2004, 2005 to 2008, 2009 to 2012, 2013 to 2016, March 2017 to 2020, and August 2021 to 2023.

### 2.2. Study population

**Figure 1.**
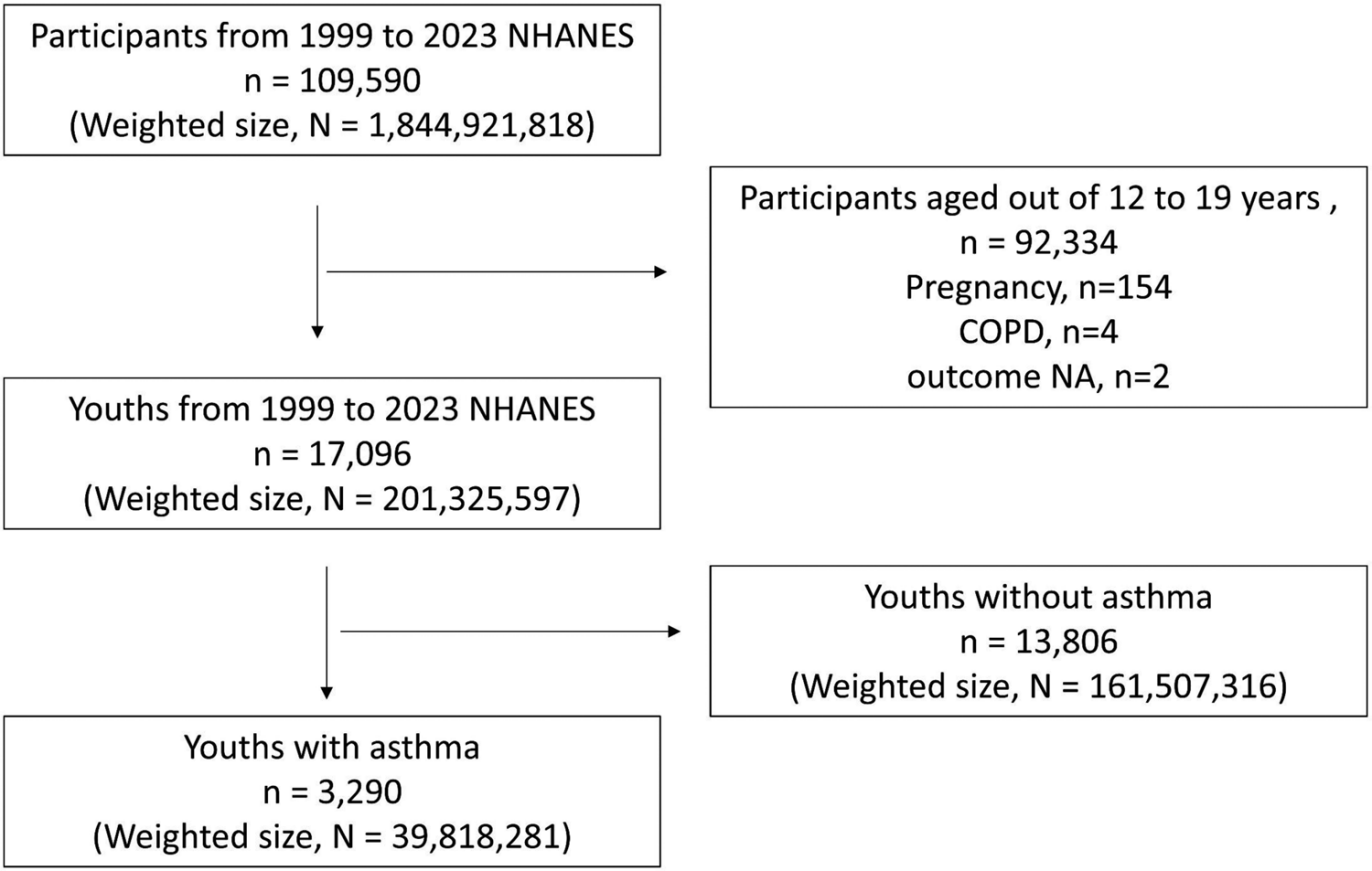
Flow chart of the selection of the study population. NA = not available, NHANES = National Health and Nutrition Examination Survey.

The total sample size after cycle combination was 17,096 adolescents aged 12 to 19 years, including 3290 with current asthma – sample size sufficient for subgroup analyses. Self-reported data may be subject to recall bias; however, asthma diagnostic criteria were based on clinical physician assessments, and attack assessments aligned with CDC-recommended definitions, reducing the risk of misclassification. Participants were excluded if they had chronic obstructive pulmonary disease, were pregnant, or had missing data for asthma status^[2]^ (Fig. 1).

### 2.3. Variable definitions

#### 2.3.1. *Asthma definition*:

Asthma was defined using NHANES core survey questions: participants were classified as having current asthma if they responded affirmatively to both “Has a doctor or other health professional ever told you that you had asthma?” and “Do you still have asthma?”

#### 2.3.2. Self-reported poor control indicators:

Self-reported poor control indicators were assessed based on 2 self-reported outcomes in the past year: experiencing an asthma attack and emergency department (ED) visits for asthma (included as a marker of severe poor control, contingent on reporting an attack) to capture clinical severity, consistent with global guidelines.^[12]^ The denominator for these analyses was the subset of participants with current asthma; those reporting either outcome were classified as having self-reported poor control indicators.

#### 2.3.3. Sociodemographic variables:

Sociodemographic variables included: Sex (male/female); Age (stratified as 12–15 years and 16–19 years); Race and ethnicity (non-Hispanic White, non-Hispanic Black, and other groups; “other” included multiracial groups as defined by NHANES); Parental educational attainment.

### 2.4. Sensitivity analysis

To address potential biases introduced by COVID-19-related disruptions (e.g., paused data collection, modified survey protocols, shifts in self-reporting behavior), a sensitivity analysis was conducted by excluding the most affected survey cycle (March 2017–2020) – consistent with NHANES guidance advising against combining this cycle with pre-pandemic periods. The sensitivity analysis replicated the primary analyses, including trend tests for asthma prevalence and rates of self-reported poor control indicators, as well as subgroup disparity assessments, using only pre-pandemic cycles (2001–2016) and the post-pandemic resumption cycle (August 2021–2023). This approach aimed to verify the robustness of the primary findings.

### 2.5. Ethics approval and informed consent

This study used de-identified public data from the NHANES, which has been approved by the National Center for Health Statistics Ethics Review Board (NCHS ERB; approval reference not publicly disclosed by NHANES for routine surveillance protocols). All participants in NHANES provided written informed consent prior to data collection, in compliance with the ethical principles outlined in the Declaration of Helsinki. Since this study analyzed preexisting, de-identified, and publicly available data without accessing any individual-level identifying information, no additional ethical approval was required for the present analysis. The use of NHANES data was conducted in accordance with the NCHS data access guidelines (https://wwwn.cdc.gov/nchs/nhanes/default.aspx).

### 2.6. Statistical analysis

All analyses were conducted using R software (version 4.4.0) with the ‘survey’ package, which incorporated NHANES primary sampling units (PSU) and stratification variables (stratum) to account for the complex sampling design. We used combined NHANES mobile examination center (MEC) and interview weights to account for sampling design, non-response, and post-stratification, as recommended by NHANES (https://wwwn.cdc.gov/nchs/nhanes/default.aspx). Variance and 95% confidence intervals (95% CI) were calculated with adjustment for clustering effects to avoid variance underestimation. Linear and quadratic terms were tested to identify nonlinear trends, with model selection based on the Akaike Information Criterion (AIC). Adjusted Wald tests were used to compare differences between subgroups, accounting for sampling weights and clustered design. A 2-sided *P* value < 0.05 was considered statistically significant. The NHANES protocol was approved by the National Center for Health Statistics Ethics Review Board, and all participants provided written informed consent.

## 3. Results

Differences between subgroups were tested using adjusted Wald tests in the survey regression models, accounting for sampling weights and clustered design. The unweighted study population included 17,096 youths (weighted mean age, 15.4 years; 48.9% female), of whom 3290 were identified as having asthma. Asthma prevalence showed a fluctuating trend, with no statistically significant difference in higher-order trend analysis (*P* = .312); tests for linear and quadratic trends (to assess nonlinearity) revealed no statistically significant trends overall (*P*>.05). Subgroup-specific analyses also showed no significant nonlinear trends, though some subgroups exhibited small fluctuations over time. The prevalence was 18.46% (95% CI, 16.89%–20.03%) in 2001 to 2004 and 20.34% (95% CI, 17.79%–22.89%) in 2021 to 2023, with no statistically significant linear trend over the entire period (*P* = .273). This small absolute change (1.7 percentage points) should not be interpreted as an increase, as the trend was not statistically significant (Table 1).

**Table 1:** Estimated prevalence of asthma among US youths by sociodemographic factors, from 2001 to 2023 NHANES.

Characteristic	Participants, No. (%)	Weight prevalence, % (95% CI) (N = 17,096)*							Difference in prevalence, % (95% CI)*		
		2001–2004 (n = 4682)	2005–2008 (n = 3479)	2009–2012 (n = 2609)	2013–2016 (n = 2748)	2017–2020 (n = 2001)	2021–2023 (n = 1577)	*P* for trend†	2021–2023 vs 2001–2004‡	2017–2020 vs 2013–2016§	2021–2023 vs 2017–2020∥
Overall		18.46 (16.89–20.03)	19.51 (17.64–21.38)	20.22 (18.42–22.02)	20.41 (18.10–22.71)	19.66 (17.38–21.94)	20.34 (17.79–22.89)	.273	1.88 (−1.11 to 4.88)	−0.75 (−3.99 to 2.50)	0.68 (−2.74 to 4.10)
Age group, yr											
12–15	8833 (51.7)	17.83 (15.75–19.92)	19.28 (17.26–21.31)	19.88 (17.05–22.70)	20.25 (17.36–23.13)	20.29 (16.90–23.68)	21.41 (17.80–25.03)	.087	3.58 (−0.59 to 7.75)	0.04 (−4.41 to 4.49)	1.12 (−3.83 to 6.08)
16–19	8263 (48.3)	19.14 (16.98–21.31)	19.75 (17.03–22.46)	20.59 (18.07–23.11)	20.59 (17.66–23.52)	19.01 (14.38–23.65)	19.03 (15.59–22.47)	.831	−0.11 (−4.18 to 3.96)	−1.57 (−7.06 to 3.91)	0.02 (−5.76 to 5.79)
Sex											
Female	8353 (48.9)	18.51 (16.68–20.34)	18.78 (16.09–21.48)	18.66 (15.68–21.63)	20.42 (17.67–23.17)	18.24 (14.37–22.12)	19.15 (16.17–22.13)	.779	0.64 (−2.86 to 4.13)	−2.18 (−6.93 to 2.57)	0.91 (−3.98 to 5.79)
Male	8743 (51.1)	18.41 (15.80–21.01)	20.21 (18.26–22.15)	21.72 (18.92–24.51)	20.39 (17.84–22.94)	21.02 (17.21–24.82)	21.49 (18.00–24.99)	.220	3.08 (−1.28 to 7.44)	0.62 (−3.96 to 5.20)	0.48 (−4.69 to 5.64)
Race/ethnicity											
Mexican American	4205 (24.6)	13.33 (11.59–15.08)	13.33 (10.80–15.86)	14.70 (11.92–17.48)	17.24 (13.50–20.98)	14.29 (10.17–18.40)	17.37 (11.03–23.71)	.172	4.04 (−2.54 to 10.61)	−2.05 (−8.51 to 2.61)	3.08 (−4.48 to 10.64)
Non-Hispanic Black	4704 (27.5)	21.68 (18.09–25.28)	19.67 (16.35–22.98)	24.76 (21.40–28.12)	28.56 (24.46–32.66)	23.43 (17.73–29.13)	24.32 (20.46–28.19)	.100	2.64 (−2.64 to 7.92)	−5.13 (−12.15 to 1.89)	0.89 (−6.00 to 7.78)
Non-Hispanic White	4994 (29.2)	18.31 (16.04–20.59)	20.27 (17.37–23.17)	20.51 (17.68–23.34)	19.52 (16.06–22.98)	20.75 (16.21–25.29)	18.03 (15.05–21.01)	.962	−0.28 (−4.03 to 3.47)	1.23 (−4.48 to 6.94)	−2.71 (−8.15 to 2.71)
Others¶	3193 (18.7)	20.05 (14.91–25.20)	21.36 (16.17–26.55)	19.65 (16.03–23.27)	19.21 (14.21–24.21)	18.57 (13.91–23.23)	23.64 (18.14–29.14)	.453	3.59 (−3.94 to 11.12)	−0.63 (−7.47 to 6.20)	5.07 (−2.14 to 12.27)

CI = confidence interval, NHANES = National Health and Nutrition Examination Survey.

* The values for percentage (95% CI) are weighted using National Health and Nutrition Examination Survey sample weights to be nationally representative and standardized to the overall sample-weighted age distribution. Prevalence values of 10% and greater are rounded to the nearest whole number.

† *P* values for trend from 2001 to 2023 are age adjusted.

‡ Indicates the absolute increase or decrease in prevalence between 2001 to 2004 and 2021 to 2023.

§ Indicates the absolute increase or decrease in prevalence between 2013 to 2016 and 2017 to 2020.

∥ Indicates the absolute increase or decrease in prevalence between 2017 to 2020 and 2021 to 2023.

¶ Owing to the small sample size, other racial and ethnic groups were not included in the analysis. “Other” included groups indicated as “other” in NHANES, including multiracial.

**Figure 2.**
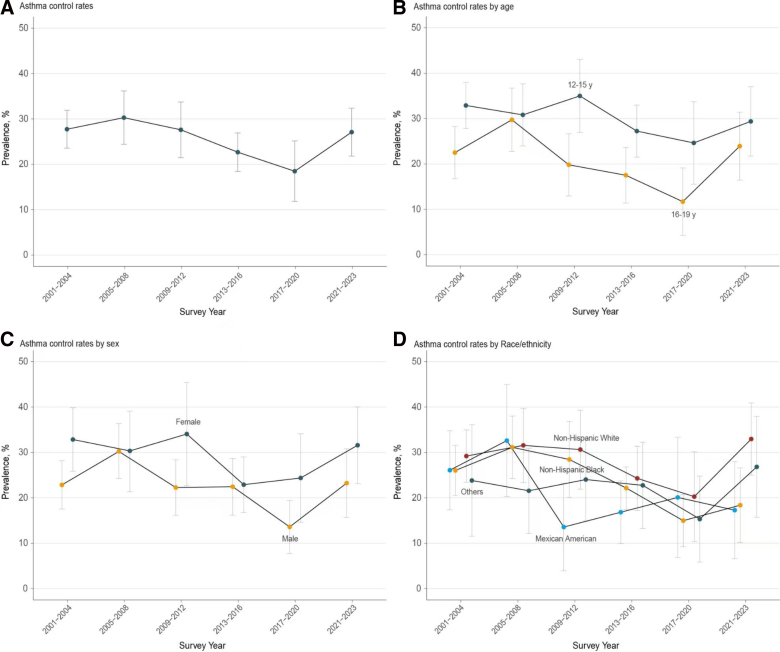
Estimated rates of self-reported poor control indicators among US youths by age, sex, and race/ethnicity, 2001 to 2023 NHANES. (A) Rates of self-reported poor control indicators among US youths in the NHANES from 2001 to 2004 to 2021 to 2023 (unweighted n = 3290). Self-reported poor control indicators was determined by the following events: asthma attacks and emergency department visits for asthma over the past year. Error bars indicate 95% confidence intervals. (B–D) show the trends in rates of self-reported poor control indicators by age, sex, and race/ethnicity. Error bars indicate 95% confidence intervals; wide intervals in the 16 to 19-yr age group reflect smaller sample sizes and reduce interpretability of subgroup differences. NHANES = National Health and Nutrition Examination Survey,

Rates of self-reported poor control indicators also fluctuated over the study period. The lowest control was observed during 2017 to 2020 (18.47% [95% CI, 11.81%–25.14%]), with a partial rebound by 2021 to 2023 (27.09% [95% CI, 21.81%–32.36%]); however, no significant improvement was detected over time (*P* = .056). Adolescents aged 16 to 19 consistently exhibited higher rates of self-reported poor control indicators compared to those aged 12 to 15, with the lowest rate occurring in 2017 to 2020 (11.69% [95% CI, 4.24%–19.13%]) (Fig. 2). Adolescents aged 16 to 19 consistently exhibited lower rates of self-reported poor control indicators compared to 12 to 15 years, though wide 95% CIs overlap substantially across age groups, limiting interpretability. Notably, the 16 to 19 years age group exhibited wider 95% CIs compared to the 12 to 15 years group, reflecting smaller sample sizes and reduced estimate precision – subgroup differences should be interpreted with caution due to overlapping CIs. Females generally showed better asthma control than males throughout the study period. Similarly, non-Hispanic White youths had higher rates of self-reported poor control indicators than non-Hispanic Black and other racial/ethnic groups. Wide 95% CIs for non-Hispanic Black and “other” racial/ethnic groups indicate limited statistical precision, and observed differences should not be overinterpreted as definitive subgroup disparities.

## 4. Discussion

Multiple national surveillance reports and epidemiological studies have documented the substantial and persistent burden of asthma across the U.S. population, with adolescent populations identified as a high-risk group for suboptimal disease management and adverse outcomes.^[2,5,12]^ Most prior research has focused on either short-term trend changes or cross-sectional disparities in adolescent asthma, with limited evidence characterizing 20 + year longitudinal patterns in both prevalence and disease control, as well as the long-term evolution of sociodemographic inequalities in this age group. Against this background, this study highlights 2 key findings regarding the asthma burden among U.S. youths aged 12 to 19 years over the 22-year study period. First, asthma prevalence remains disproportionately high among marginalized populations – particularly non-Hispanic Black adolescents – consistent with prior national epidemiological studies that have consistently identified racial and ethnic disparities in pediatric and adolescent asthma prevalence in the U.S.^[13–15]^ Notably, our study extends these prior findings by demonstrating that no statistically significant narrowing of this racial disparity gap was observed after 2012, a critical observation that is not well characterized in existing short-term surveillance reports. Second, rates of self-reported poor control indicators among U.S. adolescents have persisted with sociodemographic differences and remained suboptimal overall, with no statistically detectable improvement observed over the 22-year study period or after 2012. This finding aligns with prior research and 2025 CDC national surveillance data documenting stagnant asthma control rates among U.S. adolescents, which have long lagged behind national public health targets,^[16,17]^ while our study further provides novel evidence that this suboptimal control status has not improved over 2 decades of national asthma prevention and control efforts. The observed fluctuations in these rates across survey cycles are descriptive and do not represent statistically significant “declines” or “rebounds,” given the nonsignificant trend test results.

Compared with previously published national asthma surveillance analyses, the core contributions of this study to the existing evidence base are reflected in 3 aspects. First, this study focuses on the 12 to 19 years age group, a critical transition period for asthma management, and conducts an independent systematic trend analysis of this group based on 22-year long-cycle nationally representative data, filling the gap in existing studies regarding long-term dynamic change analysis of this subgroup. Second, this study simultaneously evaluates the long-term trends of asthma prevalence, disease control status, and sociodemographic disparities, fully presenting the full-dimensional change characteristics of asthma disease burden among adolescents from morbidity to control outcomes. Third, this study clarifies the long-term evolution of sociodemographic inequalities related to adolescent asthma in the United States, especially the change trend of disparities after 2012, which can provide solid national evidence support for the development of precise asthma prevention and control intervention strategies targeting adolescent groups and the reduction of health disparities.

All findings from the subgroup analyses in this study represent descriptive observations of between-group differences in self-reported poor asthma control indicators, rather than confirmed causal associations. Given the descriptive nature of this study, which did not include multivariate models to adjust for potential confounding factors, no definitive causal inferences can be drawn regarding the observed between-group differences. Across the 22-year study period, we observed that adolescents aged 16 to 19 years consistently had lower rates of self-reported poor asthma control indicators compared with those aged 12 to 15 years; however, the 95% confidence intervals (CIs) of the estimates for these 2 age subgroups overlapped substantially across all survey cycles, indicating that the observed between-group differences were not statistically significant and should be interpreted with caution. With regard to sex, we observed that male adolescents consistently had higher rates of self-reported poor asthma control indicators than female adolescents throughout the study period. For racial and ethnic subgroups, we observed that non-Hispanic White adolescents had higher rates of self-reported poor asthma control indicators than non-Hispanic Black adolescents and those from other racial/ethnic groups, a descriptive pattern consistent with prior epidemiological studies on adolescent asthma outcomes. However, the 95% CIs for non-Hispanic Black adolescents and other racial/ethnic groups were wide, reflecting limited statistical precision of the estimates, and the observed differences should not be overinterpreted as definitive between-group disparities. Notably, without multivariate adjustment for potential confounders, this study cannot attribute the observed between-group differences to any specific driving factors. Future studies incorporating adjusted multivariate analyses are warranted to validate the observed patterns and clarify potential causal pathways.

Three core limitations of this study should be noted. First, asthma status and self-reported poor control indicators were based on self- or proxy-reported data rather than objective clinical measures (e.g., spirometry), which may introduce recall bias or outcome misclassification. This limitation is consistent with nearly all large-scale national asthma surveillance studies using NHANES data.^[18]^ Second, this study is a descriptive cross-sectional trend analysis without adjustment for key confounders previously linked to adolescent asthma outcomes, including medication adherence, healthcare access barriers, and environmental exposures (e.g., ambient air pollution, indoor allergen exposure).^[15]^ Thus, we cannot establish causal associations between sociodemographic factors and the observed asthma outcomes, nor can we disentangle the independent drivers of the persistent disparities identified. Third, our analysis has inherent limitations related to data collection disruptions and statistical power. The COVID-19 pandemic caused interruptions to NHANES data collection in the 2017 to 2020 cycle, which may have introduced bias to the primary analysis; while our sensitivity analysis excluding this cycle confirmed the robustness of our core findings, residual impacts of the pandemic on healthcare-seeking behavior in the 2021 to 2023 cycle cannot be fully ruled out. Additionally, subgroup analyses were limited by reduced sample size and wide 95% CIs, particularly for smaller demographic subgroups, which may have reduced our ability to detect true between-group differences.

The persistence of, and no evidence of narrowing in, sociodemographic disparities in asthma prevalence and self-reported poor control indicators over 22 years—particularly among adolescents aged 16 to 19 years, males, and non-Hispanic Black youth—underscores the urgent need for targeted, equity-focused asthma interventions for this vulnerable transitional age group. Consistent with recommendations from the 2024 Global Initiative for Asthma (GINA) guidelines and prior national asthma disparity research,^[19]^ these targeted interventions should prioritize equitable healthcare access, mitigate modifiable environmental asthma risks, and advance community-based asthma management initiatives to alleviate disease management burdens in marginalized adolescent populations. Future large-scale studies incorporating multivariate adjusted analyses for key confounders and objective clinical measures of asthma control are needed to clarify the causal pathways driving the observed disparities, and to evaluate the effectiveness of targeted interventions for reducing the asthma burden among U.S. adolescents.

## Acknowledgments

This study was supported by National supercomputing Taiyuan center.

## Author contributions

**Conceptualization:** Jun Qiao.

**Data curation:** Qi Cui, Keran Jia, Jun Qiao.

**Formal analysis:** Qi Cui.

**Funding acquisition:** Liyun An.

**Investigation:** Qi Cui.

**Methodology:** Jun Qiao.

**Project administration:** Jun Qiao.

**Supervision:** Liyun An, Fukun Wang.

**Validation:** Fukun Wang.

**Visualization:** Fukun Wang.

**Writing – original draft:** Qi Cui, Zhongyan Xu.

**Writing – review & editing:** Zhongyan Xu.
